# PrEP4Her: Evaluating the Reach, Adoption, Preliminary Effectiveness and Fidelity of a Multi‐Component Hybrid Type 3 Trial Designed to Increase PrEP Prescriptions for Black Women Receiving Gynaecologic Care in a University‐Affiliated Women's Health Setting in the United States South

**DOI:** 10.1002/jia2.70208

**Published:** 2026-10-08

**Authors:** Anais Mahone, Audra Williams, Dave Cleveland, Bernadette Johnson, Grace Watson, Mirjam‐Colette Kempf, Lynn T. Matthews, Michael J. Mugavero, Latesha E. Elopre

**Affiliations:** ^1^ Division of Infectious Diseases Department of Medicine Heersink School of Medicine University of Alabama at Birmingham Birmingham Alabama USA; ^2^ Division of Women's Reproductive Healthcare Department of Obstetrics and Gynecology Heersink School of Medicine University of Alabama at Birmingham Birmingham Alabama USA; ^3^ University of Alabama at Birmingham (UAB) Center for AIDS Research, University of Alabama at Birmingham Birmingham Alabama USA; ^4^ Schools of Nursing Public Health and Medicine University of Alabama at Birmingham Birmingham Alabama USA; ^5^ School of Medicine Yale University New Haven Connecticut USA

**Keywords:** HIV prevention, implementation science, pre‐exposure prophylaxis (PrEP), sexual health, women's health

## Abstract

**Introduction:**

To address suboptimal HIV pre‐exposure prophylaxis (PrEP) uptake among Black women, we designed PrEP4Her, a multi‐phase, multi‐component implementation strategy aimed to increase PrEP prescriptions among women receiving routine health services at a university‐affiliated gynaecology (GYN) clinic located in the Southeastern United States.

**Methods:**

Informed by intervention mapping, PrEP4Her, a hybrid type 3 trial, was piloted over 8 months, January 2024–August 2024, and the Reach, Effectiveness, Adoption, Implementation, and Maintenance (RE‐AIM) conceptual framework guided the evaluation of proximal implementation outcomes. In this manuscript, we present (1) *Reach* (the number of Black women prescribed PrEP and the proportion of PrEP prescribed to women with an indication, having a sexually transmitted infection (STI) within 12 months and/or reporting >1 sex partners collected from a universal health screener); (2) *Adoption* (the number of PrEP prescribers); (3) *Preliminary Effectiveness* (PrEP prescribed over time). Also, we measured the number of Black women who self‐reported talking to a doctor about PrEP as a secondary outcome *(Fidelity)*. We collected evaluative data from the electronic health record (EHR), sexual health screener and self‐administered post‐visit survey. Descriptive statistics were utilized to describe the sample, and chi‐square tests examined the relationships between patient characteristics and talking to providers about PrEP. Analyses were completed in October 2025.

**Results:**

EHR data indicate there were 1274 unique first clinic visits. Most women identified as Black race (58%) and, on average, were 34 (±11.3) years old. Ten PrEP prescriptions were written by seven prescribers (i.e. adoption). Among women prescribed PrEP, nine identified as Black women and none had a recent STI (i.e. reach). PrEP was prescribed once before the pilot, eight times during the pilot and once in the month after the pilot. However, PrEP was not prescribed to women with PrEP indications (*N* = 85) (i.e. preliminary effectiveness). Nearly half (43%) of surveyed women reported talking to a provider about PrEP (i.e. fidelity); most (49%) identified as Black race. Chi‐square tests show significant differences by race (*p* = 0.02).

**Conclusions:**

We observed an increase in PrEP prescriptions from before to during the pilot period; however, 85 women with PrEP indications were not prescribed PrEP. Future research will further evaluate PrEP4Her's acceptability and feasibility.

## 1 Introduction

Despite biomedical advances to decrease HIV transmission, Black women continue to experience a disproportionate burden of disease. In the United States (U.S), Black women outpace all other racial/ethnic groups, representing more than 50% of HIV diagnoses among women annually [1]. HIV pre‐exposure prophylaxis (PrEP) is highly effective in reducing the risk of HIV transmission when taken [2]. Unfortunately, in 2024, only 9% of eligible women were PrEP users [3]. Suboptimal PrEP uptake is even greater for Black women, who account for 2% of PrEP users nationally. In Alabama, the PrEP‐to‐need (PNR) ratio, a measure of total PrEP users to the number of HIV diagnoses, is dismal for Black individuals (PNR = 3.4), indicating suboptimal PrEP reach in the state [4].

Complex factors influence Black women's PrEP knowledge and engagement [5, 6, 7, 8]. The socio‐cultural climate in the U.S. South, such as limited health access, racism and poverty, influences women's access to PrEP [9]. On an individual level, Black women may have limited knowledge of PrEP, and those with PrEP awareness reported hesitancy towards using the medication due to factors like underrepresentation within PrEP marketing materials [10]. Further, women may perceive that PrEP is only intended for use among gay and bisexual men or women with multiple sex partners. These views may perpetuate stigmatizing beliefs that limit women's willingness to initiate PrEP [11].

Further, provider and clinic‐level barriers limit providers’ ability to engage in PrEP discussions with women during routine medical visits; time is often cited as a primary barrier to these conversations, as providers navigate prioritization of patients’ primary reason for visit with comprehensive PrEP care [12, 13]. Practitioners also report limited PrEP knowledge, including assessment of PrEP eligibility. Some providers report that training in sexual health care or PrEP varies based on where, and when, providers received training as biomedical prevention innovations continue to advance, inhibiting their comfort and ability to disseminate PrEP information [12].

Despite these barriers, recent qualitative findings highlight that women desire to speak with providers about PrEP and feel providers are best positioned to promote HIV prevention tools to Black women during clinic visits [14, 15]. Moreover, when providers included PrEP care as a component of their workflow, PrEP discussions did not create a significant time burden [13]. Leveraging these critical patient−provider interactions to increase PrEP prescriptions requires diversifying PrEP access to non‐infectious disease clinical settings where women are more likely to receive health services. Women's health settings, like family planning and gynaecology clinics, are critical healthcare access points for PrEP initiation and appropriate settings for PrEP service delivery [14, 16, 17, 18]. Well‐designed education materials are effective tools that may support patient–provider discussions, ensuring providers feel confident disseminating PrEP information and remain informed on current PrEP innovations [12, 19].

With this knowledge, we implemented PrEP4Her, a multi‐component implementation strategy, designed to increase PrEP prescriptions among Alabama women with a focus on reaching Black women. Using the RE‐AIM framework, we evaluated reach, adoption, preliminary effectiveness and fidelity implementation outcomes for women engaged in routine gynaecologic care at a university‐affiliated women's health clinic.

## 2 Methods

### 2.1 Study Design

Formative development of the PrEP4Her implementation strategy began in 2023 when researchers utilized both qualitative (in‐depth interviews and focus group discussions) and quantitative methods (surveys) to explore clinic and provider‐level barriers to PrEP service delivery and Black women's preferences for receiving PrEP education from gynaecology providers [19]. Results from these studies were presented to two community advisory boards, one composed of Black women in Birmingham, Alabama, and one comprised of gynaecology providers. In partnership with these groups, the research team designed the multi‐component PrEP4Her implementation strategy. PrEP4Her was implemented as a single‐arm, hybrid 3 pilot trial from January 2024 to August 2024 within a university‐affiliated gynaecology clinic in Birmingham. At the time of this study, PrEP was not routinely prescribed in Alabama gynaecology clinics, meaning comparison to other sites would have limited meaning. Instead, we measured our implementation outcomes as the number of PrEP prescriptions before, during and after PrEP4Her's implementation.

The combination strategy was developed using an intervention mapping approach, and the developmental work has been described [14, 19, 20]. PrEP4Her utilized provider and patient‐facing education materials on HIV and PrEP, didactic provider trainings, an electronic health record (EHR) order set, a PrEP indications job aide, pamphlets, a website and a universal sexual health screener to increase PrEP knowledge, including PrEP eligibility. Trainings were delivered on site and asynchronously [19]. This manuscript reports: reach, adoption, effectiveness and fidelity.

### 2.2 Location/Participants

PrEP4Her was implemented at the GYN Continuity Clinic. This clinic serves approximately 17,000 women annually. Among these women, 40% identified as Black women [19]. The clinical team consists of medical assistants, nurse practitioners, licensed and registered nurses, and attending physicians. Medical residents provide clinical services and rotate in the clinic every 6 weeks. The clinic offers several sexual and reproductive health services, including routine gynaecologic care, contraceptive care and sexually transmitted infection (STI) screening and treatment [14].

All women who received care at the clinic during the study period were expected to be exposed to the PrEP4Her implementation strategy as part of their standard care. To participate in the PrEP4Her post‐visit survey, eligibility criteria included (1) assigned female at birth, (2) English‐speaking, (3) aged 18–45 and (4) confirmed to have HIV‐negative serostatus at the time of initial visit.

### 2.3 Conceptual Framework

The Reach, Effectiveness, Adoption, Implementation, and Maintenance (RE‐AIM) framework was used to evaluate three proximal implementation outcomes: (1) reach, (2) adoption and (3) preliminary effectiveness. Additionally, we measured (4) fidelity, defined as the number of Black women who self‐reported talking to their provider about PrEP, as indicated in a post‐visit survey, as a secondary outcome. Other implementation outcomes (feasibility and acceptability) will be reported in a separate manuscript. Figure 1 highlights the PrEP4Her components (tools), the change mechanisms we aimed to achieve with PrEP4Her and our study's proximal implementation outcomes.

**FIGURE 1:**
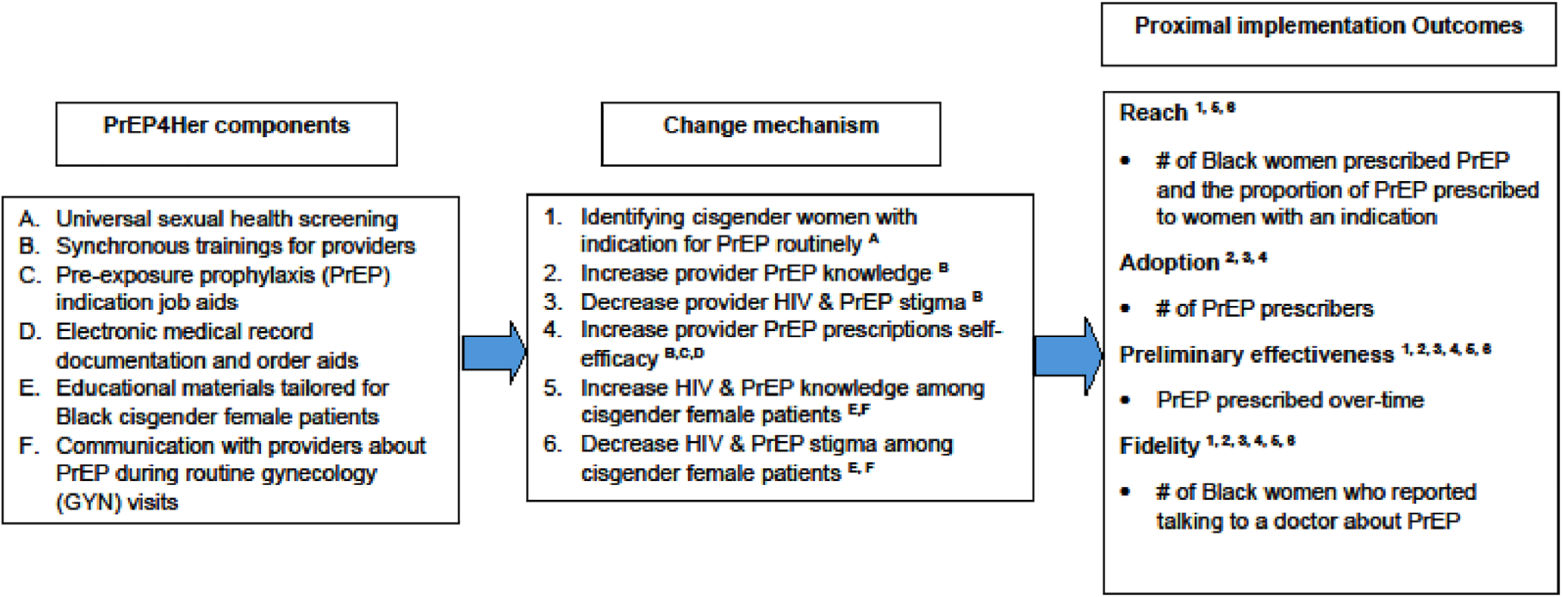
The figure displays each of the PrEP4Her components and the associated change mechanism. The proximal implementation outcomes guided evaluation of our results.

#### 2.3.1 Proximal Implementation Outcomes

##### 2.3.1.1 Reach Outcome

We defined reach as the number of Black women prescribed PrEP (primary) and the proportion of PrEP prescribed to women with an indication as documented in the EHR (secondary). Our definition of PrEP indications was based on the Centers for Disease Control and Prevention's guidance summary criteria [21]. We defined women as having a PrEP indication if they (1) were diagnosed with an STI in the last 12 months; and (2) had more than one sex partner. These indications are not complete but are those that could be defined in the EHR.

Additionally, we collected socio‐demographic characteristics to identify the number of Black women prescribed PrEP.

##### 2.3.1.2 Adoption Outcome

Adoption was measured as the number of PrEP prescriptions, per the EHR.

##### 2.3.1.3 Preliminary Effectiveness Outcome

Preliminary effectiveness was measured as the number of PrEP prescriptions prescribed over time as documented in the EHR. We calculated this number by counting PrEP prescribed during the 6‐month period before the pilot, the 8‐month pilot and during the month after the pilot.

##### 2.3.1.4 Fidelity Outcome

Using a self‐administered, post‐visit survey, we also report participants’ self‐report of discussions with their provider about PrEP during the clinic visit. Participants self‐reported their socio‐demographic characteristics.

#### 2.3.2 Data Collection

##### 2.3.2.1 EHR Data

EHR data were used to confirm the number of PrEP prescriptions, demographics of recipients, PrEP indications (STI in the past 12 months and/or more than one sex partner), the number of PrEP prescribers and prescriptions written over time (reach, adoption, preliminary effectiveness). PrEP initiation was operationalized as receipt of a PrEP prescription, formulated as tenofovir disoproxil furmrate/emtricitabine (TDF/FTC), the only PrEP product approved for use among people with vaginal exposure to HIV by the U.S. Food and Drug Administration at the time. Additionally, we screened records to confirm the medication was prescribed for HIV prevention, excluding women with an HIV diagnosis, ongoing hepatitis B treatment or post‐exposure prophylaxis. Lastly, prescriptions must have been prescribed by a site clinic provider to a patient who received care in the clinic.

#### 2.3.3 Measures

##### 2.3.3.1 Universal Sexual Health Screener

Clinic staff administered a sexual health screener, a component of the PrEP4Her implementation strategy, to assess participants’ self‐reported STI history in the last 12 months and number of sex partners. The total number of women with a PrEP indication defined the denominator for our reach implementation outcome.

##### 2.3.3.2 Post‐Visit Survey

A sub‐population of women seen in the clinic during the 8‐month PrEP4Her pilot period completed a post‐visit survey to capture the end‐user experience. The post‐visit survey was designed, completed and self‐administered using RedCap software. Women who accessed care in the clinic were invited to self‐report their socio‐demographic data, history of sexual behaviours, engagement with PrEP4Her study materials in the clinic and provider discussions about PrEP (fidelity). Survey participants were recruited using convenience sampling strategies including displaying study flyers in the clinic space and direct referrals from clinic staff. Participants were paid $25 as an incentive.

#### 2.3.4 Data Analysis

Descriptive statistics (median, standard deviations, frequencies and percentages) were used to describe the sample. We conducted chi‐square tests to examine the associations between participants’ characteristics and participants’ self‐report of talking to a provider about PrEP.

#### 2.3.5 Ethics

This study was approved by the University of Alabama at Birmingham's Institutional Review Board (IRB). Informed consent was completed and documented for all study participants.

## 3 Results

### 3.1 Participant Characteristics

Characteristics of women who accessed health services during the PrEP4Her pilot period are described based on EHR data (Table 1). Between January and August 2024, there were 1274 first clinic encounters. All women were screened for STIs and HIV. Most (58%) identified as Black, while approximately one‐quarter (26%) identified as White. On average, participants were 34 years old (± 11.3).

**TABLE 1:** Characteristics of women, by first encounter, who accessed the gynaecology (GYN) clinic for care during the PrEP4Her pilot January 2024–August 2024, electronic health record (EHR) review, *N* = 1274.

Characteristics	*N* (%)
Race/ethnicity	
Black/African American	712 (57.6)
White	327 (26.4)
Hispanic or Latino	135 (10.9)
Asian	24 (1.9)
Others	8 (0.6)
Decline	31 (2.5)
**Age (M, SD), years**	34.4 (± 11.3)
Sex, as indicated in electronic health record	
Female	1271 (99.8)
Trans	2 (0.06)
At least one positive STI screening (chlamydia, gonorrhoea, syphilis only)	
No STI	1243 (97.6)
At least one	31 (2.4)
At least one positive STI screening (including HIV)	
No STI	1241 (97.4)
At least one	33 (2.6)
Positive chlamydia screening, during pilot period	
Yes	18 (1.4)
No	1256 (98.6)
Positive gonorrhoea screening, during pilot period	
Yes	9 (0.7)
No	1265 (99.3)
Positive syphilis screening, during pilot period	
Yes	9 (0.7)
No	1265 (99.3)
Positive HIV screening, during pilot period	
Yes	2 (0.2)
No	1272 (99.8)
HIV‐serostatus negative	
Positive STI	31 (2.4)
Negative STI	1243 (97.6)
HIV‐serostatus positive	
Negative STI	2 (0.16)
Visits with PrEP indications, *N* = 555	
No indication	470 (84.7)
STI in the last 12 months	6 (1.1)
Multiple partners	78 (14.1)
STI in the last 12 months and multiple partners	1 (0.2)

Abbreviations: M, mean; PrEP, pre‐exposure prophylaxis; SD, standard deviation; STI, sexually transmitted infection.

Per the EHR, fewer than 3% (*N* = 31) of women received a positive STI screening during the pilot period. Eighteen (<2%) screened positive for chlamydia, nine (0.7%) for gonorrhoea and nine (0.7%) for syphilis. Most women had a negative HIV screening (99%). Few HIV‐serostatus‐negative women had a positive STI (*N* = 31, 2.4%). Among women with PrEP indications documented by the sexual health screener, most did not have an indication for PrEP (85%) (defined as curable STI and report of multiple sexual partners), while 14.1% of women had reports of multiple sexual partners; few women were both diagnosed with an STI in the last 12 months and had multiple partners (0.2%).

The proportion of women with PrEP indications, from our total population, was 0.15 (85/555, 15%).

### 3.2 Reach, Adoption and Preliminary Effectiveness

#### 3.2.1 Reach

We collected the characteristics of the women who received PrEP prescriptions (Table 2). Ten PrEP prescriptions were confirmed. Most women who received a PrEP prescription were Black (91%), and none of the women had a PrEP indication (positive STI diagnosis) (0%). None of these women had data suggesting multiple partners. Women prescribed PrEP were, on average, aged 30.2 (± 8.46) years.

**TABLE 2:** Characteristics of women who received a pre‐exposure prophylaxis (PrEP) prescription in a gynaecology (GYN) clinic in Alabama before, during and after the PrEP4Her pilot (July 2023–August 2024), confirmed by electronic health record (EHR) chart review, *N* = 10.

	Received a PrEP prescription during visit
Characteristics	*N* (%)
Race/ethnicity	
Black/African American	9 (91%)
Other	1 (9%)
Age (M, SD), years	30.2 (± 8.46)
Positive chlamydia, gonorrhoea or syphilis screening, during pilot period	
Yes	0 (9%)
No	10 (91%)
Positive HIV diagnosis, during pilot period	
Yes	0 (0%)
No	10 (100%)
PrEP prescription timeline	
6 months before PrEP4Her	1 (9%)
During PrEP4Her	8 (82%)
1 month after PrEP4Her	1 (9%)
Proportion of women prescribed PrEP during the pilot period	0/85 (0%)

Abbreviations: M, mean; SD, standard deviation.

PrEP was not prescribed to women with a PrEP indication (0/85, 0%).

#### 3.2.2 Adoption

Further, we leveraged the EHR to identify the number of PrEP prescribers at the GYN clinic during our study. There were seven unique prescribers.

#### 3.2.3 Preliminary Effectiveness

We defined preliminary effectiveness as the number of PrEP prescriptions prescribed over time. One prescription was initiated before PrEP4Her, eight prescriptions were initiated during the pilot and one prescription was initiated in the month after the pilot.

#### 3.2.4 Fidelity

Women who received care during the PrEP4Her pilot were invited to participate in our post‐visit survey. A convenience sample of 236 women participated. Results are presented to inform our fidelity outcome (Table 3).

**TABLE 3:** Characteristics of women who received the PrEP4Her implementation strategy as a component of their routine sexual healthcare and responded to the post‐visit survey asking whether they spoke with a provider about pre‐exposure prophylaxis (PrEP) during the pilot period from January 2024 to August 2024, *N* = 236.

Characteristics	Total sample *N* (%)	Spoke with provider *N* (%)	Did not speak with provider *N* (%)	*p*‐value
**Total**	236	108 (45%)	128 (53%)	
Race/ethnicity				0.02
Black/African American	161 (68.8%)	79 (49.1%)	82 (50.9%)	
White	54 (23.1%)	22 (40.7%)	32 (59.3%)	
Hispanic or Latino	9 (3.8%)	0 (0.0%)	9 (100.0%)	
Multiple races chosen	9 (3.8%)	6 (66.7%)	3 (33.3%)	
American Indian or Alaska Native	1 (0.4%)	0 (0.0%)	1 (100.0%)	
Missing	2			
Education				0.93
Less than college	40 (16.9%)	19 (47.5%)	21 (52.5%)	
College/graduate/doctoral degree	186 (78.8%)	84 (45.2%)	102 (54.8%)	
Prefer not to answer/missing	10 (4.2%)	5 (50.0%)	5 (50.0%)	
Missing	0			
Employment status				0.79
Full‐time/self‐employed	84 (37.2%)	40 (47.6%)	44 (52.4%)	
Part‐time/unemployed/student/retired/cannot work	142 (62.8%)	65 (45.8%)	77 (54.2%	
Missing	10			
Insurance				0.33
Private/Medicare/CHIP/military or other gov	37 (15.7%)	17 (45.9%)	20 (54.1%)	
Medicaid/self‐pay	187 (79.2%)	88 (47.1%)	99 (52.9%)	
Prefer not to answer/missing	12 (5.1%)	3 (25.0%)	9 (75.0%)	
Household income				0.75
Less than $4500	69 (33.8%)	36 (52.2%)	33 (47.8%)	
$4500 to < $30,000	88 (43.1%)	39 (44.3%)	49 (55.7%)	
$30,000 to < $50,000	37 (18.1%)	17 (45.9%)	20 (54.1%)	
$50,000 or more	10 (4.9%)	4 (40.0%)	6 (60.0%)	
Missing	32			
Reason for visit				0.23
Routine annual visit	67 (28.4%)	36 (53.7%)	31 (46.3%)	
Contraceptive care	21 (8.9%)	6 (28.6%)	15 (71.4%)	
Multiple reasons	53 (22.5%)	24 (45.3%)	29 (54.7%)	
Other	95 (40.3%)	42 (44.2%)	53 (55.8%)	
Missing	0			
Number of partners				0.26
1	142 (60.2%)	71 (50.0%)	71 (50.0%)	
More than one	49 (20.8%)	20 (40.8%)	29 (59.2%)	
Missing	45 (19.1%)	17 (37.8%)	28 (62.2%)	
Vaginal or anal sex in the last 6 months				0.25
Yes	196 (83.1%)	93 (47.4%)	103 (52.6%)	
No	40 (16.9%)	15 (37.5%)	25 (62.5%)	
How often used condoms during sex				0.055
Some of time	70 (76.9%)	30 (42.9%)	40 (57.1%)	
All of time	21 (23.1%)	14 (66.7%)	7 (33.3%)	
Missing	132			
Gonorrhoea or syphilis diagnosis, 6 months				0.26
Yes	4 (2.0%)	3 (75.0%)	1 (25.0%)	
No	192 (98.0%)	90 (46.9%)	102 (53.1%)	
Missing	40			
Partner living with HIV and unmedicated				0.94
No	194 (99.0%)	92 (47.4%)	102 (53.1%)	
Unsure	2 (1.0%)	1 (50.0%)	1 (50.0%)	

Abbreviation: CHIP, Children's Health Insurance Program.

We conducted chi‐square tests of significance to examine the relationship between sample characteristics and whether women spoke to a provider about PrEP for a subset of women (*N* = 236) who completed our post‐visit survey.

Most women in the survey identified as Black/African American (69%). Nearly half (45%) reported talking to their provider about PrEP (fidelity). Comparatively, White women accounted for one‐quarter of the total sample, and among this group, 41% of women reported speaking with a provider about PrEP. Among *N* = 10 Hispanic, American Indian or Alaska Native women, none reported speaking with a provider about PrEP. However, there were more multi‐racial women (*N* = 9) who reported talking to a provider about PrEP (6, 67%) compared to one‐third of women in this group who did not. Race/ethnicity had notable results, indicating a relationship between racial identity and speaking to a provider about PrEP (*p* = 0.02).

Approximately one‐quarter of women (28.4%) were seen for a routine annual visit. A higher percentage of women in this group (53.7%) reported speaking to a provider about PrEP. Most women reported having one sexual partner (60.2%), and *N* = 71 (50%) in this group reported talking to a provider about PrEP. Among women reporting more than one sexual partner (49, 21%), 41% reported talking to a provider about PrEP.

Most women reported having vaginal or anal sex within the last 6 months prior to participating in this survey (83.1%), and nearly half of these women spoke to a provider about PrEP (47%). Less than a quarter (23.1%) of women used condoms all of the time. More women in this group reported talking to a provider about PrEP (compared to those who did not (66.7% vs. 33.3%, *p* = 0.055).

## 4 Discussion

To our knowledge, PrEP4Her was among the first multi‐component, implementation science strategy designed and piloted to increase PrEP prescriptions for Black women receiving services at a university‐affiliated gynaecology clinic in Alabama, located in the Southeastern United States. We observed an increase in the number of PrEP prescriptions from one prescription in the period before the PrEP4Her pilot to eight prescriptions during the pilot period, although 85 women with PrEP indications were still missed and not prescribed PrEP. Our post‐survey results indicate almost half (45%) of women talked to a provider about PrEP and showcased significant outcomes in the association between race/ethnicity and whether women talked to their provider about PrEP (*p* = 0.02), indicating differences in PrEP discussions by racial identity (fidelity).

Our results mirror outcomes of a PrEP intervention that leveraged clinical decision support tools and a registered nurse counsellor to increase PrEP prescriptions among cisgender women served in an academic medical centre in the northeastern US. While this clinical trial implemented similar tools such as patient‐focused PrEP education materials and provider orders, and, innovatively, utilized EHR alerts to identify patients with an elevated HIV acquisition risk (indicated by a signed antibiotic prescription for an STI), the intervention similarly resulted in low PrEP prescription rates [22]. Further, although our study and the previously mentioned trial reported increased PrEP discussions and counselling, these interactions did not translate to substantive PrEP initiations. However, these outcomes may indicate that PrEP counselling and discussions are key features in increasing PrEP awareness, notably in our study where statistically significant differences in PrEP discussions by race should be further explored.

While our pilot implemented clinic staff trainings only prior to the PrEP4Her pilot, a recent study aimed to increase PrEP prescriptions among women in Washington, DC in the United States by implementing ongoing provider education, which resulted in increased providers’ reported confidence in PrEP discussions (i.e. adoption). Further, the combination of provider‐level training, EHR alerts and patient‐facing PrEP education materials in the clinic resulted in increases in PrEP offering to PrEP‐eligible women, increased PrEP initiation, and more than half of women remaining on PrEP at their 3‐month follow‐up. These results are poignant for future iterations of PrEP4Her, particularly when considering how we can increase provider‐level training to ensure resident physicians are prepared to discuss and prescribe PrEP despite the frequent staff rotations [23, 24]. This strategy, in addition to a larger period of study, can improve PrEP prescribing during the study and into the maintenance period.

Further, while results are mixed, we may consider use of EHR alerts to help providers identify PrEP‐eligible women, especially as emerging research points to use of machine learning algorithms to predict HIV acquisition risk. This is especially relevant as few machine learning HIV acquisition risk strategies are designed for assessing solely women [22, 25, 26]. As we continue to examine strategies to increase PrEP engagement, our pilot makes a meaningful contribution to the literature by demonstrating the use of sexual health screeners and educational tools to integrate PrEP care as a standard component of women's gynaecological care. This is forward movement along the PrEP cascade for this population in this setting.

This study has some limitations. Although PrEP4Her's implementation should have exposed clinic patients to some component of the combination strategy (i.e. sexual health screener, talking with a provider about PrEP, educational materials in the clinic space), we were unable to confirm what component was most effective for end users or how often participants engaged with each component. In the future, we will consider how to measure end‐user engagement, strategies to ensure screeners reach all patients and continue to provide on‐site and asynchronous training to staff, focusing on medical residents, considering their short clinic rotations. To meet this need, we will identify training modalities that fit the needs of residents to receive PrEP knowledge and ensure tools such as the EHR order set and job aides are accessible. Further, we are uncertain if other factors occurred during the PrEP4Her pilot that contributed to the increase in PrEP prescriptions or facilitated patient‐provider PrEP discussions. We did not have a control group to compare outcomes; however, we can report that there were no other trials ongoing during PrEP4Her's implementation. We also had a small sample size, and surveys were administered only at one point, without any participant follow‐up.

The GYN providers were engaged in PrEP4Her. In the future, we may consider utilizing support staff, like medical assistants, as PrEP champions. As evidenced in our clinic site, these professionals often engage with the participants first and for the longest duration of time [27]. Further, we understand that the use of convenience sampling for the post‐pilot survey may have introduced selection bias. Our participants may have had an interest in HIV prevention and PrEP discussions or PrEP initiation. It is possible that women receiving care in an urban area may have had more exposure to PrEP education materials or care from other health providers. Also, the women who visited the clinic during the PrEP4Her study, overall, had low STI incidence and few reported multiple partners during the last 12 months; therefore, our findings are not generalizable to populations of women who may have higher HIV acquisition risk. This study did not examine differences between the overall population of women receiving care and the women who consented to participate in our survey. We also acknowledge the potential for social desirability bias, which may have impacted women's survey engagement or may have resulted in under‐reporting of sexual health behaviours due to stigma.

Despite these limitations, we piloted PrEP4Her among an ending the epidemic (EHE) priority population of women, who were all screened for STIs and HIV. Further, PrEP4Her successfully reached a large population of Black women. Our study efforts are translatable to international contexts by showcasing the potential strategies to increase PrEP prescriptions and conversations with women through population‐specific education and marketing in non‐infectious disease settings.

## 5 Conclusions

Our study contributes to a growing area of research examining and evaluating PrEP prescribing implementation strategies for women's health settings. PrEP4Her specifically addressed the needs of Black women in the Deep South, a group experiencing multi‐level and complex barriers to HIV preventative care. Future studies should consider EHR tools to capture objective measures of PrEP prescribing and increasing provider‐level training to ensure, notably, resident physicians are well‐prepared to engage women in PrEP discussions.

## Author Contributions

LEE conceptualized the study and acquired funding for this project. AW conceptualized the study. DC supported data analyses for this study. LTM, M‐CK and MJM advised on the project investigation and methodology, analysis and interpretation of findings. BJ and GW performed activities associated with administration and data collection. AM wrote and edited the manuscript and contributed to data analyses. All authors provided feedback on the manuscript.

## Funding

This study was supported by National Institutes of Health (Grants R34 MH128002/MH/NIMH NIH HHS/United States).

## Conflicts of Interest

LEE served as an investigator on a Merck Pharmaceuticals research grant.

## Data Availability

Research data are not shared due to ethics and privacy concerns.

## References

[1] CDC , Fast Facts: HIV and Women. *HIV*, https://www.cdc.gov/hiv/data‐research/facts‐stats/women.html (2025, accessed 30 October 2025).

[2] Conversation Starters , Start Talking. Stop HIV , http://www.cdc.gov/actagainstaids/campaigns/starttalking/convo.html (2025, accessed 30 October 2025).

[3] AIDSVu Releases 2024 PrEP Use Data Showing Growing Use Across the U.S. AIDSVu, https://aidsvu.org/news‐updates/aidsvu‐releases‐2024‐prep‐use‐data‐showing‐growing‐use‐across‐the‐u‐s/. (2025, accessed 30 October 2025).

[4] P. S. Sullivan , S. N. DuBose , A. D. Castel , et al., “Equity of PrEP Uptake by Race, Ethnicity, Sex and Region in the United States in the First Decade of PrEP: A Population‐Based Analysis,” Lancet Regional Health‐Americas 33 (2024), 10.1016/j.lana.2024.100738. PMC11041841 38659491

[5] J. Troutman , A. Robillard , L. A. Ingram , et al., “Individual, Social, and Structural Vulnerability for Black Women in the South: Implications for PrEP,” AIDS Education and Prevention 35 (2023): 290–308. 10.1521/aeap.2023.35.4.290 37535327

[6] W. C. Irie , A. Mahone , R. Heffron , et al., “Where Do We Go From Here? Reconciling Implementation Failure of PrEP for Black Women in the South. Leveraging Critical Realism to Identify Unaddressed Barriers as We Move Forward,” Frontiers in Reproductive Health 6 (2024): 1449554, 10.3389/frph.2024.1449554. PMC11611828 39628794

[7] A. Chory and K. Bond , “Access to PrEP and Other Sexual Health Services for Cisgender Women in the United States: A Review of State Policy and Medicaid Expansion,” Frontiers in Public Health 12 (2024): 1360349. 10.3389/fpubh.2024.1360349 PMC11231431 38983260

[8] F. Smit and T. B. Masvawure , “Barriers and Facilitators to Acceptability and Uptake of Pre‐Exposure Prophylaxis (PrEP) Among Black Women in the United States: A Systematic Review,” Journal of Racial and Ethnic Health Disparities 11 (2024): 2649–2662. 10.1007/s40615-023-01729-9 37531021

[9] L. A. Nydegger , J. Dickson‐Gomez , and T. K. Ko , “Structural and Syndemic Barriers to PrEP Adoption Among Black Women at High Risk for HIV: A Qualitative Exploration,” Culture, Health & Sexuality 23 (2021): 659–673. 10.1080/13691058.2020.1720297 PMC7529643 32212993

[10] M. J. Hill , S. Sapp , S. McCants , et al., “An Examination of Perceptions Among Black Women on Their Awareness of and Access to Pre‐Exposure Prophylaxis (PrEP),” International Journal of Environmental Research and Public Health 21 (2024): 1084. 10.3390/ijerph21081084 PMC11354061 39200693

[11] L. M. Hill , O. Allison , O. Adeniran , et al., “PrEP Knowledge and Perceptions Among Women Living in North Carolina Public Housing Communities,” PLoS ONE 20 (2025): e0320093. 10.1371/journal.pone.0320093 PMC11957289 40163424

[12] A. Caplon , K. A. Alexander , T. Kershaw , et al., “Assessing Provider‐, Clinic‐, and Structural‐Level Barriers and Recommendations to Pre‐Exposure Prophylaxis (PrEP) Uptake: A Qualitative Investigation Among Women Experiencing Intimate Partner Violence, Intimate Partner Violence Service Providers, and Healthcare Providers,” AIDS and Behavior 25 (2021): 3425–3436. 10.1007/s10461-021-03297-7 PMC8419149 33974167

[13] C. Zhang , W. Chang , and Y. Liu , “Understanding the PrEP Care Continuum for Adults: Health Care Providers' Perspectives on Barriers, Facilitators, and Missed Opportunities,” AIDS Patient Care and STDs 39 (2025): 61–69. 10.1089/apc.2024.0241 39873657

[14] M. C. Pratt , H. Goymer , K. Burgan , et al., “Qualitative Analysis of Patient and Key Informant Interviews to Inform Integration of HIV Pre‐Exposure Prophylaxis Services Into Gynecology Care in Alabama,” Womens Health 21 (2025): 17455057251331714. 10.1177/17455057251331714 PMC12062644 40339168

[15] A. I. Sophus , J. W. Mitchell , J. Barroso , et al., “Factors Associated With Planned Future Use of PrEP in the Next 3 Months and Likelihood to Use PrEP Among Black Cisgender HIV‐Negative Women in Texas,” AIDS and Behavior 28 (2024): 72–92. 10.1007/s10461-023-04188-9 37768428

[16] S. K. Butler , Y. Kislovskiy , K. Bunge , et al., “Implementing Pre‐Exposure Prophylaxis for HIV Prevention in Women: The Role of the Obstetrician‐Gynecologist,” American Journal of Obstetrics and Gynecology 226 (2022): 764–772. 10.1016/j.ajog.2021.12.263 34973180

[17] C. M. Khosropour , E. Healy , E. M. Murphy , et al., ““I Honestly Didn't Know It Was for Women Too…” Acceptability and Feasibility of Integrating PrEP Services Into OB/GYN Practices From the Perspectives of Practice Staff and Cisgender Women in the Community: A Qualitative Study,” Frontiers in Health Services 5 (2025): 1567688, 10.3389/frhs.2025.1567688. PMC12162616 40521174

[18] L. Elopre , C. Mcdavid , B. Johnson , et al., “P420 Understanding PrEP Service Delivery Preferences Among Black Women in Urban and Rural Counties in the US Deep South,” Sexually Transmitted Infections 95 (2019): A200–A201.

[19] M. Sohail , L. Matthews , A. Williams , et al., “Improving Pre‐Exposure Prophylaxis Provision as Part of Routine Gynecologic Care Among Black Cisgender Women (Project PrEP4Her): Protocol for the Implementation of an Intervention,” JMIR Research Protocols 14 (2025): e58976. 10.2196/58976 PMC11953599 40085134

[20] H. Goymer , M. Pratt , K. W. Burgan , et al., “P‐505. Integrating PrEP Services Into a Gynecology Clinic in Alabama: A Qualitative Analysis of Key Determinants,” Open Forum Infectious Diseases 12 (2025): ofae631.704.

[21] Clinical Guidance for PrEP, https://www.cdc.gov/hivnexus/hcp/prep/index.html (30 April 2026).

[22] R. Wang , T. F. Fruhauf , S. S. Sao , et al., “Clinic‐Based Interventions to Increase Preexposure Prophylaxis Awareness and Uptake Among United States Patients Attending an Obstetrics and Gynecology Clinic in Baltimore, Maryland,” American Journal of Obstetrics and Gynecology 229 (2023): 423.e1–423.e8. 10.1016/j.ajog.2023.07.046 37531988

[23] L. H. Izadi , O. Mmeje , E. F. Drabo , et al., “An Effectiveness‐Implementation Trial Protocol to Evaluate PrEP Initiation Among U.S. Cisgender Women Using eHealth Tools vs. Standard Care,” Frontiers in Reproductive Health 5 (2023): 1196392, 10.3389/frph.2023.1196392. PMC10285440 37361343

[24] F. Silvestri , N. Alvarez , O. Afzal , et al., “Training Obstetrician Gynaecologists in HIV PrEP (Pre‐Exposure Prophylaxis): A 2‐Year Experience,” Sexually Transmitted Infections 99 (2023): 348–350. 10.1136/sextrans-2022-055691 37015803

[25] M. E. Clement , J. Thomas , C. Kelsey , et al., “HEalth Record Optimization for Identifying Candidates for HIV PRe‐Exposure Prophylaxis: A Community‐Informed Approach to Model Development,” AIDS Patient Care and STDs 39 (2025): 266–272. 10.1089/apc.2025.0022 PMC12259409 40576614

[26] J. L. Marcus , L. B. Hurley , D. S. Krakower , et al., “Use of Electronic Health Record Data and Machine Learning to Identify Candidates for HIV Pre‐Exposure Prophylaxis: A Modelling Study,” Lancet HIV 6 (2019): e688–e695. 10.1016/S2352-3018(19)30137-7 PMC7152802 31285183

[27] K. N. Piper , C. Escoffery , J. M. Sales , et al., “Models of HIV Pre‐Exposure Prophylaxis Care Used in Title X Family Planning Clinics in the Southern U.S,” Journal of Adolescent Health 68 (2021): 480–487. 10.1016/j.jadohealth.2020.10.005 PMC7902302 33160826

